# Better Lunch Boxes: Testing the Feasibility and Acceptability of a Family-Based Pilot Intervention to Support Nutritious Home-Packed Lunches

**DOI:** 10.3390/children12060739

**Published:** 2025-06-06

**Authors:** Tamara Petresin, Jess Haines, Danielle S. Battram, Virginie Desgreniers, Ivanna Regina Pena Mascorro, Claire N. Tugault-Lafleur

**Affiliations:** 1 Department of Family Relations and Applied Nutrition, University of Guelph, Guelph, ON N1G 2W1, Canada; petresit@uoguelph.ca (T.P.); jhaines@uoguelph.ca (J.H.); 2 Brescia School of Food and Nutritional Sciences, Western University, London, ON N6G 2V4, Canada; dbattra@uwo.ca; 3 School of Nutrition Sciences, University of Ottawa, Ottawa, ON K1N 6N5, Canada; vdesg080@uottawa.ca (V.D.); ipena093@uottawa.ca (I.R.P.M.)

**Keywords:** family-based intervention, feasibility study, school-aged children, packed lunches, Canada

## Abstract

**Background/Objectives:** The majority of Canadian children bring a home-packed lunch to school, and previous research suggests lunches are of poor nutritional quality. This pilot study aimed to test the feasibility, acceptability, and preliminary impact of an eHealth family-based intervention designed to improve the nutritional quality of home-packed lunches. **Methods**: In this 12-week intervention, families (n = 20 parents with children aged 4–8 years) received a toolkit which included a cookbook on tips for preparing healthy lunches and 15 tested lunch box-friendly recipes, a lunch box, text messages, and an online cooking class. Feasibility was assessed via documentation of intervention delivery and participant retention rates. Acceptability was assessed via post-intervention surveys and semi-structured interviews in a sub-sample of parents (n = 9). Preliminary impact was assessed using 3-day lunch food records. Descriptive statistics were used to assess feasibility and acceptability, and Wilcoxon signed-rank tests were used to evaluate changes in the nutritional content of packed lunches. **Results**: Findings indicated a high retention rate (85%), and the majority (94%) of participants reported that the intervention was helpful and that they would recommend it to another parent. Qualitative interviews suggest parents found the recipes practical and diverse, the lunch box and the cooking class helpful, and some reported increased confidence and greater awareness of the foods being packed. No changes in the nutritional content of packed lunches were observed (n = 10 children). **Conclusions**: In summary, a home-packed lunchbox intervention is feasible and well accepted by families, but further refinements are needed to optimize its impact before a full-scale trial.

## 1. Introduction

The foods Canadian children consume at school have recently come into the spotlight, with research showing that lunches are of poor dietary quality [1,2,3,4]. In 2015, Canadian children reported consuming about one-third of their total daily calories at school, but reported relatively fewer vegetables, whole grains, and milk and alternatives during school hours compared to the remainder of the school day [2,3]. In addition to not consuming enough foods in line with Canadian dietary guidelines, lunch boxes include excessive amounts of minimally nutritious foods such as sugary drinks and salty packaged snacks, as they are often perceived as convenient and shelf-stable [2,5]. Similar nutrient compositions have been observed in lunch boxes across the globe, including in Australia [6], the UK [7], and the US [8]. Globally, these studies demonstrate a need for effective interventions to support the provision of nutritionally balanced lunchboxes.

In Canada, there is no nationwide school lunch program [9]. Although national surveillance of children’s eating habits in Canadian schools remains inconsistent, prior analyses suggest that on a given school day, approximately three-quarters of children bring a home-packed lunch to school [10]. While a National School Food Policy was released in 2024 [11], there is still a long road ahead before the implementation of such a program. To improve the dietary intake of Canadian children at school, complementary interventions targeting multiple stakeholders (families and school staff) are needed.

Parents often face various challenges when it comes to packing healthy lunches, including time constraints, a lack of menu ideas, child preferences, food safety concerns, and managing food allergies or other dietary needs [12,13,14,15,16]. Furthermore, qualitative evidence underscores that mothers often feel judged for the food choices they make for their children and lack accessible guidance on what constitutes appropriate school lunches [17]. Together, these findings emphasize the need for interventions that provide practical support to empower parents in overcoming these diverse challenges.

Evidence from school- and childcare-based interventions highlights several strategies that can support families in packing healthier lunches. A 2019 systematic review and meta-analysis found that programs emphasizing parental education and practical tools, such as nutrition resources and web-based interventions, have had some success in increasing parental provision of vegetables [18,19]. These interventions often include multicomponent web-based resources and offer practical activities to involve children in lunch packing and help shift parents’ attitudes and intentions around healthy lunch packing [19,20,21]. In Canada, limited studies have explored supporting parents in packing nutritious home-packed lunches. Previous research has shown that providing families with a nutrition education booklet with healthy lunch ideas and tips for involving children in lunch preparation was well-received by parents, although they expressed a desire for more applied learning opportunities [22]. Similarly, recent interventions targeting household food waste reduction and plant-based protein intake demonstrated that applied learning opportunities like cooking classes, educational messages received via text messages, tangible tools like kitchen items, and a cookbook with child-friendly recipes were feasible and well-received by families with school-aged children [23,24].

Acceptable and feasible interventions are needed to improve the quality of foods children bring to school, and to our knowledge, no family-based intervention has been conducted in the Canadian context. Feasibility studies are a necessary first step before the roll-out of a full-scale efficacy trial. Therefore, our primary aim was to assess the feasibility and acceptability of the Better Lunch Box program, a multicomponent family-based intervention that aimed to support parents with young children (age 4–8 years) in packing nutritious lunches. Our secondary aim was to evaluate the preliminary impact on the nutritional content of foods packed.

## 2. Materials and Methods

### 2.1. Study Design, Participants, and Study Procedures

A single-arm, pre-post design was used to evaluate the feasibility and acceptability (primary outcomes) and preliminary impact of the intervention. Families were recruited from January to March 2024 (mid-way in the school year) using social media advertisements and sent recruitment flyers via email to distribution networks. Potential participants were invited to complete an online screener questionnaire to evaluate eligibility. Inclusion criteria included living in one of three Ontario cities: Ottawa, Guelph, or London, having at least one parent who could respond to surveys in English and receive text messages, and having a child in Junior Kindergarten to Grade 3 (which corresponds to ages 4 to 8 years). We excluded parents with advanced nutrition/culinary training or whose children participated in a school lunch program more than 3 days per week. A quota sampling approach was used to recruit families from diverse socioeconomic backgrounds, including at least 30% of parents who did not have university/college-level education. Eligible parents were asked to complete a consent form and fill out a registration and baseline survey.

Web-based surveys were used to assess intervention reach (e.g., sociodemographic characteristics of parents) and dose received (i.e., acceptability of the intervention). Parents were also asked to fill out a 3-day lunchbox food record at pre- and post-intervention to collect data on the nutritional content of the lunch boxes. Finally, participants were invited to participate in a qualitative interview to provide more in-depth data regarding their experience. Participants who filled out the survey and submitted the lunch food records received a CAD $25 grocery store gift card as an incentive at baseline and again at post-intervention (up to a total of CAD $50 in gift cards). Participants who completed the interview received a CAD $25 grocery store gift card at the conclusion of the interview.

### 2.2. Ethics and Registration

**Figure 1:**
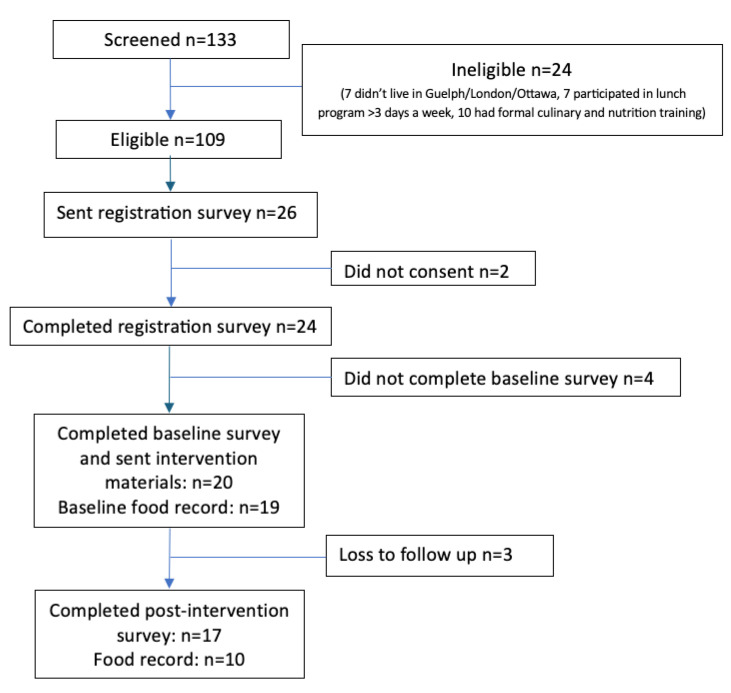
CONSORT flow diagram.

Ethics approval for this research was obtained from the University of Guelph Research Ethics Board (REB#23-06-008), the University of Ottawa Research Ethics Board (REB#H-05-23-8478), and Brescia University College Research Ethics Board (REB#2023-05-30_Battram_Danielle). The trial was not registered, but it follows the CONSORT reporting guidelines for pilot studies [25]. Figure 1 below details the participant study flow.

### 2.3. Intervention Description

The Better Lunch Box intervention was developed in consultation with parents, Registered Dietitians, and behavior change experts. The intervention was informed by the Theory of Planned Behaviour [26] and was designed to target the attitudes and perceived behavioural control related to healthy lunch packing. We targeted parents of younger children (ages 4–8 years, from Junior Kindergarten to Grade 3) since in Ontario, this corresponds to a time in which parents (most often mothers) face increasing food-related responsibilities as children transition from early education and care settings (where meals and snacks are provided) to school in which school meal programs are scarce or nonexistent. A multicomponent format focused on practical components was chosen as these have been shown to be effective in promoting healthy lunch packing [12,18,19]. An overview of the intervention components is provided in Supplemental File S1. Briefly, the components included a printed copy of the Better Lunchbox cookbook, a watertight Bento-style lunch box, biweekly text messages for four weeks, and an online cooking class led by a Registered Dietitian. The toolkit incorporated elements from formative work performed by Petresin and Battram [22] on the Healthy Lunch Box Booklet. The Better Lunch Box cookbook included: benefits of a healthy lunch, healthy lunch box food choices from each food group, tips on food safety, and how to involve children in lunch packing. Finally, the cookbook included 15 tested recipes developed with culinary experts at George Brown College’s Culinary Institute. Three team members attended two in-person taste testing sessions to ensure the recipes were nutritious, tasty, and child-friendly. After brainstorming lunch box-friendly recipe ‘concepts’ with the culinary experts, two rounds of recipe tasting were conducted to narrow down to fifteen lunch box recipes. The watertight plastic lunch box was provided to families as a tangible reinforcement to support the packing of various food groups. Biweekly text messages for four weeks were sent to parents to keep them engaged and encourage them to apply the tips and recipes from the Better Lunch Box cookbook. Finally, parents and children were invited to attend an online cooking class led by a Registered Dietitian, a graduate student in nutrition, and a school-aged child who was not participating in the intervention. The online cooking class was meant to encourage parents to apply the tips and recipes from the cookbook. Recruitment for families began in January 2024, and the intervention ran from February to June 2024 (middle to end of the school year).

### 2.4. Measures

#### 2.4.1. Feasibility, Acceptability, and Perceived Impact (Primary Aim)

Process evaluation data were collected to assess the feasibility and acceptability of the intervention. Feasibility of the intervention was assessed by considering reach, dose, and fidelity. Recruitment logs were used to determine intervention reach. Dose of the intervention was assessed by documenting attendance at the online cooking class and the number of cookbooks and texts received per family. Fidelity was assessed by ensuring each participant received all components of the intervention as intended, verifying that the program was delivered consistently across all participants. The feasibility of our measurement methods (i.e., surveys and lunch food records) was evaluated based on their completion rates.

Adapted from the formative work by Petresin and Battram (2023) [22], acceptability was measured in the post-intervention survey. Survey items asked parents to report on the number of recipes they had tried, their satisfaction with various intervention components, and their perception of the impact of the intervention (e.g., “Did you feel like the intervention changed your {child’s name} lunch box?”). Semi-structured qualitative interviews were used in a sub-sample of parents (n = 9) to get a more in-depth perspective on aspects of the intervention they liked, opportunities for improvement, as well as the perceived impact of the intervention. Interviews were conducted by two research assistants with training in qualitative interviewing. A copy of the interview guide can be found in Supplemental File S2. Interviews were all audio- and video-recorded using MS Teams and transcribed verbatim.

#### 2.4.2. Nutritional Content of Packed Lunches (Secondary Aim)

Parents were asked to fill out 3-day lunch food records along with digital pictures of the lunch box at baseline and post-intervention. Food records allowed for a more detailed description of some of the food items that might not have been visible, while digital photos enabled the team to validate information from the food records, both of which have been previously validated for estimating the content of home-packed lunches in the Canadian context [5]. Photos and details from the food records were manually coded by a research assistant (an undergraduate dietetic student) to derive food group reference amounts (“CFG servings”) for each child before and after the intervention. The 2007 Eating Well with Canada’s Food Guide food group classification was used [27]. Briefly, packed foods were coded as vegetables and fruit, total grain products (whole grains and non-whole grains), whole grains, milk and alternatives (including fortified soy-based beverages), and meat and alternatives [27]. No reference amount was coded for “other foods” (foods outside of the four food groups above, such as fats and oils, condiments, chocolate and candies, sugary and sweet treats) since there are no standards or reference amounts for these foods. However, we calculated the proportion of lunch boxes containing these “other foods” and examined the most commonly packed “other foods” from the following sub-food groups: baked goods and pastries, granola and snack bars, chocolate and candies, fats and condiments (mayonnaise, cream cheese) and chips and French fries. A random sample of 25% of food records was coded by a second research assistant to ensure an 80% inter-reliability between coders. Mean food group servings were estimated over two to three days (depending on the number of available food records) to estimate usual food group intake for each participant before and after the intervention.

### 2.5. Data Analyses

Descriptive statistics (e.g., means, frequencies, and proportions) were used to describe process outcomes. To identify themes among open-ended responses in surveys, inductive content analysis [28] was used. Template analysis, a type of thematic analysis, was used to generate themes and subthemes using an inductive approach [29,30] for the qualitative interviews. Data from the qualitative interviews were analyzed collaboratively by three members of the research team. Two members conducted preliminary coding of the data, organized emerging themes into clusters, defined an initial coding template, and finalized the coding template to provide an understanding of the parents’ experiences. Any coding discrepancies were resolved through discussion with the principal investigator. Wilcoxon signed-rank tests were used to assess changes in the amounts of food groups packed from baseline to post-intervention. IBM SPSS Statistics (Version 29) was used to complete quantitative analyses.

## 3. Results

### 3.1. Participant Characteristics

Table 1 describes demographic characteristics of participants. Parents were a mean age of 37.5 years, all identified as women, 19/20 were married or in a domestic partnership, and 16/20 identified as White.

### 3.2. Primary Outcomes: Feasibility, Acceptability, and Perceived Impact

A total of 20 parents completed the baseline assessment, and 17/20 (85%) completed post-intervention surveys. Out of 20, 19 (95%) and 10/20 (50%) completed 3-day lunch food records at baseline and post-intervention, respectively. All participants reported accessing all resources provided and reported reading, on average, 85% of the cookbook. All text messages were delivered to participants, but one family opted out of receiving messages over the course of the intervention. Nine families (9/17, 53%) attended the online cooking class. Out of the eight families who stated they did not attend a virtual cooking class, six reported watching the recording of the class that was shared with participants (6/8, 75%). For those who did not attend the cooking class or watch the recording (n = 2), reasons included family obligations and a lack of time.

Ninety-four percent (16/17) of parents found the program helpful and said they would recommend the Better Lunch Box program to another parent (16/17; 94.1%; Table 2). Parents reported the cookbook as the most helpful part of the program (13/17; 76.5%), followed by the Bento lunch box (11/17, 64.7%), the cooking class (4/17, 24.5%), and the text messages (3/17, 17.6%). Most parents agreed that the cookbook was easy to understand (16/17; 94.1%), visually appealing (17/17, 100%), and appropriate in length (16/17; 94.1%). However, most parents (12/17; 70.6%) reported trying between one to three recipes (out of the 15 from the cookbook).

A detailed description of themes and subthemes that emerged from the semi-structured interviews can be found in Supplemental File S3. Overall, parents enjoyed the Better Lunch Box intervention, appreciated the practicality and diversity of recipes provided, the quality of the lunch box, and the opportunity to participate in the cooking class. One parent shared, “It was really a good experience for us, and we really enjoyed being part of it”. Parents provided useful suggestions for improvement. One sharing that it would be a good idea to include more guidance on suggested portions of foods for the lunch box as “The one thing I found when I was ready to go is I got stumped on, ‘oh ok, the portions, where am I going to put what in the Bento box?’”. Other suggestions included including more kid-friendly activities as a complement to the cookbook to increase child engagement and providing a larger lunchbox for older participants. Some parents also expressed challenges with engaging their children in trying the provided recipes, sharing, “I just unfortunately for whatever reason couldn’t get my kids to be inspired by many of those recipes”. In regard to the text messages, some participants enjoyed receiving the text messages, while others felt they were already following the tips and did not need reminders. In terms of the cooking class, participants shared how they enjoyed being able to share the experience with their child and other families, with some sharing that it may have been even more valuable in person. While some participants stated the food record was easy to fill out, it did take a long time to record all foods, and suggested only using digital pictures with some written notes to make it less time-consuming. Finally, parents reported feeling more confident in their meal preparation, and were inspired to make different and healthier choices, sharing “I was inspired by the ideas to, you know, include like chicken wraps or fish wraps now, before my kids wouldn’t even consider fish and my son loves it now”. Parents also shared an increased level of awareness beyond what goes into the lunch box, highlighted the importance of including their child in the process, as well as that their kids are more willing to try different foods. One parent shared that “It definitely made me more aware of what I’m going to put in her lunches … it did also make me reflect on how important it is to have her help with packing her lunches and making choices”.

### 3.3. Secondary Outcomes: Nutritional Content of Lunch Boxes

Analyses included data from 10 children who submitted lunch food records at both baseline and post-intervention (Table 3). There were no differences in amounts of vegetables, whole fruit, fruit juice, non-whole grains, whole grains, milk, and alternatives. Servings of meat and alternatives decreased by 0.3 from baseline to post-intervention (*p* = 0.047). “Other foods” were prevalent in children’s lunch boxes both at baseline (in 28/29 lunches analyzed) and post-intervention (26/29 lunches analyzed). The most consumed “other foods” at baseline and post-intervention were baked goods and pastries (pre = 41.4%, post = 51.7%), granola and snack bars (pre = 31%, post = 37.9%), and chocolate and candies (pre = 24.1%, post = 27.6%).

## 4. Discussion

In this single-arm, pre-post pilot study, we found that the Better Lunch Box program was feasible and well-accepted by parents. Parents indicated high satisfaction through surveys and interviews, discussing the enjoyment of the cookbook, lunch box, text messages, and cooking class. The intervention was implemented as intended, had high retention rates, and was overall well accepted by parents. However, the use of recipes within the cookbook was limited, and few families participated in the virtual cooking class. Regarding this study’s secondary aim (preliminary efficacy), no changes were detected with respect to the foods being packed, which may be due to the small sample size and challenges experienced with dietary data collection.

This intervention demonstrated feasibility with an attrition rate of 15%, which is notably lower than the 38–50% reported in previous online nutrition-related interventions [31]. All participants (n = 20 families) accessed the resources provided (cookbook, lunch box, text messages) and found the cookbook and the Bento-style lunchboxes as the most helpful resources. However, our findings suggest that engagement with the recipes was low: the majority (70%) of families reporting trying one to three recipes (out of the 15) provided in the cookbook. Regrettably, our post-intervention survey did not ask families if they perceived the recipes as too difficult or not sufficiently kid-friendly. This information would have helped to understand the key barriers to preparing the recipes, given that parents reported that the cookbook was a helpful resource. The majority of parents interviewed reported finding the recipes realistic and simple, however, some parents highlighted that the ingredients could have been more affordable, that some ingredients were unfamiliar, and that they were unable to engage their children in trying the provided recipes, highlighting the need for changes in recipe development to better accommodate diverse cultural backgrounds, household incomes, and children’s preferences. While the cookbook and the lunch box were the most helpful components of the intervention, parents who participated in the live cooking sessions found them beneficial. However, the virtual live cooking sessions were the least used component of our intervention, with a little less than half of the families attending the cooking class. Similar to research conducted by Lu et al. [32] on in-person nutrition education classes, parents noted barriers to attending the cooking class related to family obligations and busy schedules. Families who provided suggestions for improving the program proposed offering more classes, and some suggested a preference for in-person family cooking sessions. Previous research has found that participants participating in virtual food skills programs have experienced less peer-to-peer engagement in the online environment [33], while those attending in-person have found an increased sense of community with other attendees and children [32]. In-person and online cooking classes seem to face similar barriers to participation due to personal and familial obligations, and future home-packed lunch interventions should consider offering an in-person cooking class to increase parent engagement and social connection.

Regarding the feasibility of collecting lunch food records, our findings highlight challenges related to collecting dietary data for foods that are eaten in the school context. Although some parents reported it was easy to complete the lunch food records, only 10/19 families completed the lunch food record at post-intervention despite receiving three reminders. This indicates that the food record method may have been too burdensome for participants and/or not worth the financial incentive. These findings echo previous research showing that food records or diaries pose a high burden on participants, limiting their feasibility to assess food consumption in this setting [34,35]. Our study participants suggested using only photos and visual methods, which research has found may improve cooperation and accuracy in children’s dietary assessment [36]. Further methodological research is needed to help lower participant burden on food diary methods, including ways of improving the accuracy of digital methods.

No significant differences were noted in the nutritional composition of participants’ lunch boxes (a secondary outcome) before and after the intervention. Our findings regarding the content of lunch boxes reflect ongoing challenges regarding the overall dietary quality of foods being packed in school lunches. Previous Canadian research [2,5,37,38] suggests healthy food choices such as whole fruit, vegetables, whole grains, and milk and alternatives continue to be underrepresented in children’s school lunches, although small gains in some dietary components such as whole fruit have been observed from 2004 to 2015 [3]. Our findings from this small sample suggest that most families were already including whole fruits, grain products, and some milk and alternatives in their packed lunches, suggesting some alignment with Canada’s Food Guide. However, vegetables were underrepresented, and some subgroups, such as whole grains, were almost absent. Moreover, highly processed foods such as baked goods and pastries, granola and snack bars, chocolate, and candies were omnipresent in children’s lunches. Trends in the consumption of processed foods among US and Canadian children and adolescents have increased [39,40], with processed foods accounting for almost half of children’s total daily energy intake [41]. Several factors contribute to the prevalence of highly processed foods, including convenience and time constraints that many parents face when preparing meals. This additional work required to prepare food from scratch can contribute to feelings of time scarcity and lead to time-saving behaviors, including the purchase of processed foods [42]. Parental stress, coupled with financial constraints, can reduce the time and energy available for meal preparation [43]. At the household level, addressing the challenges of time, stress, and the myriad of barriers parents (most often mothers [17,43]) face when lunch-packing [12,13,14] is needed through strategies that are tailored to address these practical challenges faced by families.

Taken together, findings from this feasibility trial offer several actionable insights for refining the intervention. First, given that the majority of participants reported only trying one to three recipes, as well as qualitative feedback expressing challenges with ingredients, affordability, and children’s engagement, suggests the need to co-develop future recipes with families and children to ensure greater appeal and relevance. Adjusting the cookbook to include flexible, simpler recipes (e.g., more substitutions, or mix-and-match components) may also enhance usability across families. Second, while the cooking class was well-received by participants who attended, it was the least-used component of the intervention. Nonetheless, some parents suggested during interviews that they would have liked a face-to-face class instead. To address this, future iterations could incorporate a face-to-face cooking class for increased engagement. Third, several parents suggested including more kid-focused materials and activities to enhance child engagement throughout the intervention. This could include interactive lunch-packing challenges, activities designed specifically for children (e.g., magnetic memo-boards, colouring pages, word searches, stickers). While the intervention primarily targeted parents, some participants reported involving their children in lunch preparation. One parent specifically noted that the program made them realize the importance of including their children in food preparation, recognizing it as an opportunity to support their child’s learning and engagement with food. This insight aligns with Canada’s Food Guide, which encourages involving children in cooking and food decisions as a way to build food literacy and foster healthy eating habits from an early age [44]. Involving children in meal preparation may also foster a sense of autonomy and increase their willingness to try new foods [45,46]. Future iterations of this intervention could further support parents in engaging children through age-appropriate food activities, potentially enhancing dietary behaviours and promoting positive mealtime experiences. This intervention did not address specific food presentation techniques (e.g., playful shapes); however, previous research has shown that the appearance appeal of fruits and vegetables improves children’s willingness to try disliked produce and can increase vegetable consumption [47]. While food presentation techniques may be a potentially useful strategy for increasing children’s interest in lunch contents, it is important to balance these recommendations with the realities of limited time and busy schedules of families. Emphasizing visually appealing or “perfect” lunchboxes can unintentionally increase parental mental load and stress, and lead to food inequities among children, particularly for families experiencing food insecurity [48,49]. Research shows that lunchbox aesthetics are often tied to societal expectations of “good parenting”, especially for mothers, which can contribute to feelings of guilt, anxiety, and undue pressure [17]. Future programs should be mindful of these challenges and aim to support all families with inclusive, practical, and realistic strategies. Together, these findings provide clear direction for refining the intervention and enhancing its feasibility in preparation for a full-scale trial.

To our knowledge, the Better Lunch Box program is one of the first Canadian interventions specifically designed to support parents of school-aged children in packing healthier home-packed lunches. Unlike many nutrition interventions that focus solely on knowledge-building, this program offered tangible, practical supports, including a lunchbox, cookbook, text messages, and a virtual cooking session to reduce barriers and improve the quality of lunches. Importantly, this pilot unveiled a novel insight: the day-to-day food-related care and responsibilities Canadian families face when packing lunches, which often go unacknowledged in public health nutrition research. These findings point to the need to co-design future strategies with parents and children to ensure that interventions are both practical and responsive to families’ diverse needs [50].

When interpreting our findings, there are important study limitations that should be considered. First, our intervention was conducted midway through the school year, which may have contributed to the lack of family engagement with parents, perhaps lacking motivation to apply and use the resources provided, with the summer break approaching. Previous research has highlighted the role of the “fresh start effect”, a psychological phenomenon where individuals are more likely to take action at times that feel like “new beginnings” can act as a catalyst for tackling health behaviour [51,52]. Although we did not experience difficulties with recruitment, it is possible that our timing in delivering the intervention led to decreased interest and engagement with the intervention content among families. Second, our sample was also comprised of mostly White participants and may not be generalizable to other racial/ethnic samples. Future interventions should make efforts to recruit a more ethnically diverse sample to provide insights into the intervention’s applicability across different populations.

Strengths of our study included engagement with parents to provide novel insight into the potential of online interventions to support home-packed, nutritious lunch-packing. The mixed methods design facilitated a holistic understanding of the intervention’s feasibility and acceptability, as well as the relevance of the intervention for research participants. Our quota sampling approach also allowed us to recruit families from diverse socioeconomic backgrounds, adding to the generalizability of the study results.

## 5. Conclusions

This study demonstrates that family-based interventions are well accepted and feasible in the Canadian context, but further refinements are needed to promote engagement with aspects of the intervention. For example, some aspects of the toolkit need to be modified, such as adding intervention components for children and co-designing simpler lunch box recipe ideas with parents. Further research is also needed to test the acceptability of in-person family cooking sessions. Once refined, future research should employ a randomized controlled design among a larger, more diverse sample of families to assess the intervention’s effectiveness in improving lunch-packing behaviours and outcomes.

## Figures and Tables

**Table 1:** Demographic characteristics of study participants (n = 20 parents).

Characteristic	
Gender, n	
Women	20
Age, mean (SD)	37.5 (3.6)
Study Region, n	
London	6
Guelph	8
Ottawa	6
Education, n	
Bachelor’s degree, n	6
College or non-university certificate or diploma	5
University degree above bachelor’s degree	4
High school graduate	4
Some high school	1
Born in Canada, n	
Yes	16
No	4
Length of Time in Canada, n	
1 to 5 years	1
6 to 10 years	3
Relationship Status, n	
Married or in a domestic partnership	19
Single (or never married)	1
Ethnicity/Race, n	
White	16
South Asian	2
Black	1
West Asian	1
Middle Eastern	1
First Nations, Inuit, Metis	1
Latino/a/x	1
Employment Status, n	
Employed full-time (at least 35 h per week)	14
Not employed, not looking for work	3
Employed part-time (less than 35 h per week)	2
Not employed, looking for work	1

**Table 2:** Acceptability of the Better Lunch Box program among parents, n = 17.

Variable	Response Options	Participants n (%)
Overall found the program helpful	Yes	16 (94.1)
	No	1 (5.9)
	Prefer not to answer/Did not answer	3 (15.0)
Most helpful program components	Cookbook	13 (76.4)
	Bento-style Lunch Box	11 (64.7)
	Family Cooking Class	4 (23.5)
	Text Messages	3 (17.6)
Cookbook was easy to understand	Strongly Agree	10 (62.5)
	Agree	6 (37.5)
	Neither Agree nor Disagree	1 (6.2)
Cookbook was visually appealing	Strongly Agree	10 (62.5)
	Agree	7 (43.7)
Cookbook was an appropriate length	Strongly Agree	9 (56.2)
	Agree	7 (43.7)
	Neither Agree nor Disagree	1 (6.2)
Number of recipes tried	1 to 3	12 (70.6)
	4 to 6	4 (23.5)
	Prefer not to answer	1 (5.8)
Recommend to another parent	Yes	16 (94.1)
	Prefer not to answer/Did not answer	1 (5.9)

**Table 3:** Changes in food group amounts before and after the intervention (n = 10).

	Pre-Intervention	Post-Intervention	Change	*p*-Value ^1^
	Mean ± SD	Mean ± SD		
Canada’s Food Guide [27] Food Group, Servings				
Vegetables	0.7 ± 0.4	0.5 ± 0.5	−0.2	0.401
Whole fruit	1.7 ± 0.9	1.6 ± 1.0	−0.1	0.838
Fruit juice	0.4 ± 0.6	0.3 ± 0.6	−0.2	0.180
Non-whole grains	1.4 ± 0.8	1.6 ± 1.2	0.1	0.959
Whole grains	0.2 ± 0.4	0.0 ± 0.1	−0.2	0.080
Milk and alternatives	1.0 ± 0.9	0.7 ± 0.6	−0.3	0.575
Meat and alternatives	0.5 ± 0.4	0.2 ± 0.2	−0.3	0.047

Foods and beverages were classified into main food groups and subgroups using the framework of the 2007 Eating Well with Canada’s Food Guide [27]. ^1^ *p*-value from the Wilcoxon signed-rank test testing for differences in mean amounts between baseline and post-intervention.

## Data Availability

The datasets analyzed for the current study are not publicly available due to ethical restrictions related to the consent given by participants at the time of study commencement. An ethically compliant dataset may be made available by the corresponding author on reasonable request and upon approval by the University of Guelph, the University of Ottawa, and Western University Research Ethics Boards.

## Supplementary Materials

The following supporting information can be downloaded at: https://www.mdpi.com/article/10.3390/children12060739/s1, File S1: Intervention Components; File S2: Interview Guide; File S3: Themes.

## Abbreviations

The following abbreviations are used in this manuscript:

CFG	Canada’s Food Guide
