# Supplementary material for: Better Lunch Boxes: Testing the Feasibility and Acceptability of a Family-Based Pilot Intervention to Support Nutritious Home-Packed Lunches
Source: Children (Basel). 2025 Jun 6;12(6):739. doi: 10.3390/children12060739 (PMC12190890; doi:10.3390/children12060739)
Supplement: Supplementary file 1 [file children-12-00739-s001.zip › BLB_Supplemental File S1.pdf]

Better Lunch Box Intervention Contents

Contents

Watertight Bento Lunch Box 2

Better Lunch Box Cookbook 3

Text Messages 5

Cooking Class 6

Watertight Bento Lunch Box

As part of the intervention, each participating family received one Bentgo™ Kids Chill Lunch Box - a colourful, durable, and kid-friendly container designed to keep school lunches fresh and appealing. This lunch box features multiple compartments to encourage balanced meals, a removable ice pack to keep food cool, and a leak-resistant design. A photo of the lunch box is provided below.

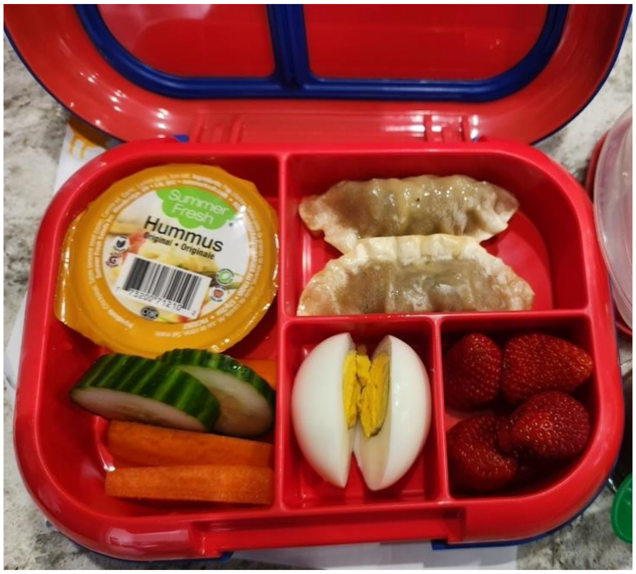

Better Lunch Box Cookbook

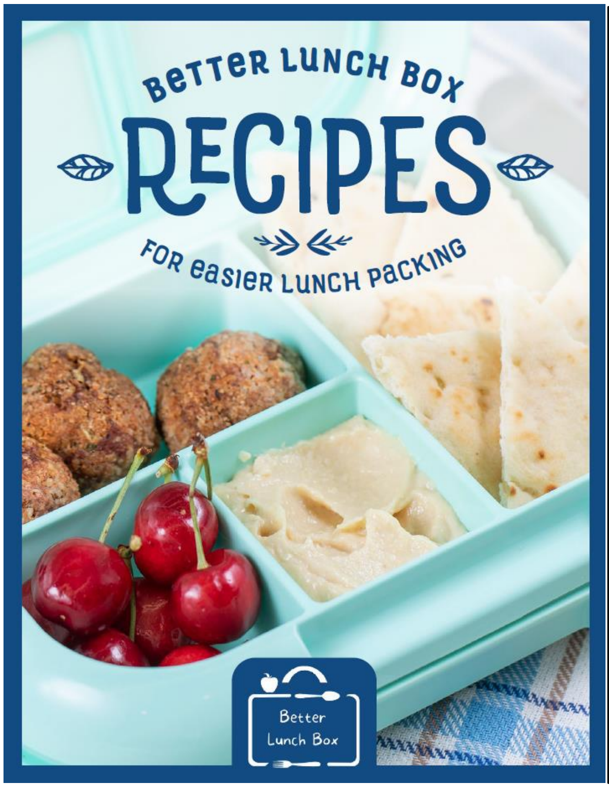

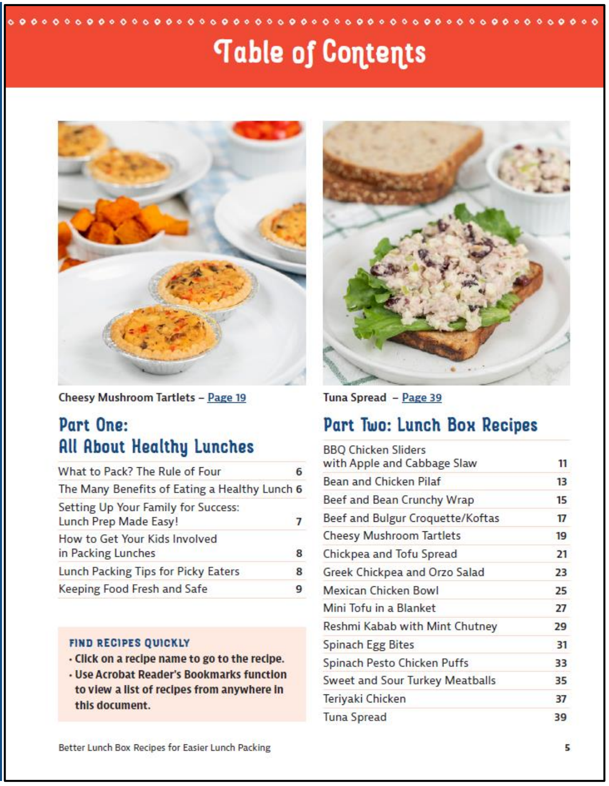

Table of Contents

Cheesy Mushroom Tartlets – Page 19

Tuna Spread – Page 39

Part One:
All About Healthy Lunches

What to Pack? The Rule of Four	6
The Many Benefits of Eating a Healthy Lunch	6
Setting Up Your Family for Success: Lunch Prep Made Easy!	7
How to Get Your Kids Involved in Packing Lunches	8
Lunch Packing Tips for Picky Eaters	8
Keeping Food Fresh and Safe	9

FIND RECIPES QUICKLY
- Click on a recipe name to go to the recipe.
- Use Acrobat Reader's Bookmarks function to view a list of recipes from anywhere in this document.

Part Two: Lunch Box Recipes

BBQ Chicken Sliders with Apple and Cabbage Slaw	11
Bean and Chicken Pilaf	13
Beef and Bean Crunchy Wrap	15
Beef and Bulgur Croquette/Koftas	17
Cheesy Mushroom Tartlets	19
Chickpea and Tofu Spread	21
Greek Chickpea and Orzo Salad	23
Mexican Chicken Bowl	25
Mini Tofu in a Blanket	27
Reshmi Kabab with Mint Chutney	29
Spinach Egg Bites	31
Spinach Pesto Chicken Puffs	33
Sweet and Sour Turkey Meatballs	35
Teriyaki Chicken	37
Tuna Spread	39

Better Lunch Box Recipes for Easier Lunch Packing 5

To support families in preparing nutritious, appealing, and practical school lunches, each family received a copy of the cookbook. The cookbook featured 15 easy-to-make recipes, offering enough variety for a three-week rotating lunch menu. Recipes varied in composition, including options that were vegetarian and culturally diverse. Each recipe was designed to be lunchbox-ready, balanced, and suitable for children's tastes, while also incorporating components from Canada's Food Guide.

Recipe Examples:

Mexican Chicken Bowl

Prep Time: 15 minutes
Cook Time: 10 minutes
Servings: 6

Ingredients:

1 lb	(454 g)	Chicken thighs, boneless and skinless, diced
1 tsp	(5 mL)	Paprika
½ tsp	(2 mL)	Salt
2 cloves		Garlic, chopped

1 tbsp	(15 mL)	Lime juice
2 tbsp	(30 mL)	Olive oil

Salsa

½ cup	(125 mL)	Black beans, drained and rinsed
½ cup	(125 mL)	Corn
½ cup	(125 mL)	Tomatoes, diced
½ cup	(125 mL)	Avocado, diced
1 tbsp	(15 mL)	Lime juice
1 tbsp	(15 mL)	Olive oil
2 sprigs		Cilantro, chopped (optional)
Pinch		Salt
Pinch		Black pepper

Method:

1. In a medium sized bowl, toss together chicken thighs, paprika, salt, garlic and 1 tbsp (15 mL) lime juice.
2. In frying pan, heat 2 tbsp (30 mL) olive oil over medium- high heat. Add seasoned chicken and sauté until chicken is cooked and internal temperature reaches 165˚F (74˚C).
3. In separate bowl, mix together black beans, corn, tomatoes and avocado.
4. Add remaining lime juice, remaining olive oil, cilantro (if using), salt and pepper. Toss well to coat.
5. Serve chicken and salsa with cooked brown rice.

Chef's Tips:

- When not in season, use frozen or canned corn.
- Coat avocado well with salsa dressing to keep the vibrant green colour.
- For older kids, try adding cumin and pickled jalapenos.

Chickpea and Tofu Spread

Prep Time: 10 minutes
Servings: 8

Ingredients:

½ lb	(227 grams)	Medium tofu, drained
1 can		Chickpeas, drained and rinsed
2 tsp	(10 mL)	Salt

¼ tsp	(1 mL)	Cayenne (optional)
¼ cup	(60 mL)	Mayonnaise
1 ½ tbsp	(22 mL)	Dill, chopped
2 tbsp	(30 mL)	Dill pickles, chopped

Method:

1. Place tofu, chickpeas, salt, cayenne and mayonnaise in blender. Pulse until spread gets to your desired consistency.
2. Remove from blender and stir in chopped dill and pickled jalapeno.

Chef's Tips:

- To turn this spread into a veggie dip, use a softer tofu. Serve with carrots, peppers or cucumbers.
- For a spicier dip, use pickled jalapeno instead of dill pickles.
- Get creative! If you like Indian flavours, try curry powder. For a Mexican dip, try pickled jalapenos and smokey chipotle powder.

Text Messages

Eight text messages were sent to parents on a biweekly basis over four weeks. The text messages are included below.

1. Struggling with ideas? Review the Better Lunch Box Recipes for Easier Lunch Packing with your child and choose a new recipe to try this week.
2. Have a picky eater on your hands? Involving your child in lunch preparation can encourage them to eat the food they've helped to pack.
3. Cook once, eat twice! Leftovers make great lunches the next day. Freezing leftovers in lunch size portions can save time and help to add some variety to lunchboxes.
4. Kids in the kitchen! Having your child help with lunch preparation helps them learn about healthy food choices, learn food skills and (most importantly!) gets them excited about eating healthy foods.
5. Why not prepare tomorrow's lunch as you do today's dinner? Pack as much as you can of your child's lunch the night before to save time in the morning.
6. Eat the rainbow every day! Vegetables and fruits are an important part of a healthy diet. Be sure to pack a variety of vegetables and/or fruits every day.
7. Planning makes a difference on busy school mornings. Making a few days' worth of sandwich fillings and cut-up veggies can save valuable time!

8. Although convenient, pre-packaged lunch kits are high in sodium and low in fibre. Pack whole foods and avoid processed foods to give your child the nutrition they need to stay focused all day.

Cooking Class

As part of the *Better Lunch Box* pilot intervention, we hosted an online interactive cooking class for families using Zoom. This 30-minute workshop was designed to promote hands-on learning, healthy eating, and family engagement in meal preparation, with a focus on creating a simple and nutritious school lunch option.

Workshop Objectives:

- Engage families in a fun, collaborative cooking experience.
- Demonstrate how to prepare the Greek Chickpea and Orzo Salad – a plant-based, versatile, and lunch box-friendly recipe.

Facilitators:

- *TP,* Registered Dietitian and Better Lunch Box research team member
- *MA*, MSc in Nutrition and passionate home cook
- *EO*, child co-facilitator

Structure & Content:

- Welcome and Icebreaker: Families were welcomed, facilitators introduced themselves, and participants were invited to share their favourite lunch foods and where they were joining from.
- Workshop Overview: Learning goals were reviewed, and facilitators emphasized the value of cooking and eating together, drawing on Canada's Food Guide recommendations.
- Recipe Introduction: The Greek Chickpea and Orzo Salad was introduced, highlighting its nutritional benefits, flexibility, and ease of preparation. Ingredients and tools were reviewed interactively, with time given for families to gather what they needed.
- Live Cooking Demonstration: Facilitators led a step-by-step walkthrough of the recipe, providing tips for child involvement and encouraging real-time interaction through questions, progress sharing, and cooking trivia.
- Wrap-Up: Families tasted their creation together, were given storage tips, and thanked for participating.

This cooking class served as a key component of the intervention by modeling healthy lunch ideas, strengthening food skills, and supporting positive mealtime routines at home.
