# Supplementary material for: Better Lunch Boxes: Testing the Feasibility and Acceptability of a Family-Based Pilot Intervention to Support Nutritious Home-Packed Lunches
Source: Children (Basel). 2025 Jun 6;12(6):739. doi: 10.3390/children12060739 (PMC12190890; doi:10.3390/children12060739)
Supplement: Supplementary file 1 [file children-12-00739-s001.zip › Supplemental file S2_Interview Guide.pdf]

INTERVIEW GUIDE – Better Lunch Box Program

Please tell us about your experience with the Better Lunch Boxes program.

On a scale of 1-10 with 1 being terrible and 10 being great, how would you rate the Better Lunch Boxes program?

- Please tell us more about why you selected that number?
- If less than 10, ask "What do you think could be done so that you would rate it a 10?"

What did you think about the Better Lunch Boxes Cookbook? [Then prompt with quality of the recipes, variety, content of the nutrition tips and education messages, etc.]

How did you feel about the quality of recipes available in the cookbook?

How did you feel about the variety and quantity (number) of recipes in the cookbook?

- Were the recipes in the cookbook relevant to your cultural and dietary preferences?

How helpful were the text messages that you received as part of the Better Lunch Box program?

- Did you follow any of the tips?
- How did you feel about the number of text messages you received?

What did you think about the Lunch Box that was provided?

- Did your child use it and did they find it easy to use/open?
- Did you find it durable? Easy to clean? Convenient to pack?

How would you describe your experience with the family cooking session/class?

- Was the cooking session an appropriate length of time?
- Was it easy to follow?
- What did you most like about it?
- What did you not like about it?
- If they did not attend, ask → Did you watch the recording of the cooking class that was sent to you? Why/why not?

What impact, if any, did the Better Lunch Boxes program have on packing healthy lunches for your child(ren)?

What was your experience like filling out the food record?

- What changes could we make to the food record to make it easier to fill out?
- Would a larger incentive be helpful to fill out the food record?

What would you change about the Better Lunch Box program to improve it for families?

Do you have any other things you would like to tell us about the Better Lunch Boxes program?
