# Supplementary material for: Better Lunch Boxes: Testing the Feasibility and Acceptability of a Family-Based Pilot Intervention to Support Nutritious Home-Packed Lunches
Source: Children (Basel). 2025 Jun 6;12(6):739. doi: 10.3390/children12060739 (PMC12190890; doi:10.3390/children12060739)
Supplement: Supplementary file 1 [file children-12-00739-s001.zip › Supplemental file S3_Interview themes.pdf]

Common themes from qualitative interviews with participants (n=9)

Themes	Subthemes	Illustrative quotes
Acceptability of the Better Lunch Box intervention	Overall satisfaction	“It was really a good experience for us, and we really enjoyed being part of it.” -Participant#6<br><br>“I think it's a great program what you guys are doing, and I think it's gonna touch on it and help a lot of families to maybe get on track with something they've been wanting to do for a long time, cause I've been kicking myself saying the same thing” -Participant #8<br><br>“I appreciate this very much actually and I have to say, I absolutely love it, I love the idea of it.” -Participant #10<br><br>“Now they're starting to try salads. So, I do thank the Lunchbox experience for that, for just kind of having the visual inspiration and the follow-ups and the reminders really, I think contributed to that effort.” - Participant #10<br><br>“I think you guys got everything right. Like you've provided help you know, the gift certificate to kind of give that motivation to go to the grocery store get the groceries, the recipe book is amazing, you've given the tools, the lunch box, bento box is great.” -Participant#10
	Recommendations for improvement	“The one thing I found when I was ready to go is I got stumped on, “oh ok, the portions, where am I going to put what in the Bento box?”-Participant#10<br><br>“A little bit more information on what kind of lunch box we can have that has separate ingredients and still fulfill their nutrient, nutrition and needs. So, let's say, I know there needs to be a protein, but like a little bit more examples as to types of and a visual of it, would probably help a little bit more and I think that's what was mainly missing for us.” - Participant #6<br><br>“Have more of a kids package just as part of the program and so that way it's easier for the parents to get them out involved. It gives assistance to the parents to get the kids involved whereas now it was just like well

		we'll trying to motivate her at the beginning or whatever on her own." - Participant #6<br><br>"I felt like I want to be there to just try with you guys and just taste what was cooking in your kitchen maybe… So being in person [the cooking class] rather than doing it on the screens, I think it would be more fun to be in person." - Participant #9<br><br>"More affordable ingredients." - Participant #1<br><br>"I think incorporating more of.. different variety of foods like replacements." - Participant #9
Acceptability of intervention components	Cookbook	"It was lovely, it was not overwhelming. I would say maybe if you make it like for little kids per se, I will get more images or thing that show the kids doing per se, to make it a little bit more interactive." - Participant #4<br><br>"It was a good book, lots of good recipes, easy, simple recipes, which is good. The kids seemed pleased with them, which is also good." -Participant #5<br><br>"I thought it was good, it was definitely like realistic, which I always appreciate, too. It's not like one of those regular cookbooks you get where you're like "Oh my gosh, I can't, that's too many ingredients", right? I thought it was good." - Participant #11<br><br>"Some good vegetarian options, meat options. That's one thing we're always looking for "protein", how to get protein in lunches, especially when we can't just rely on peanut butter these days. I thought there was a lot of really good protein options, which is awesome." - Participant #18<br><br>"I just unfortunately for whatever reason couldn't get my kids to be inspired by many of those recipes." – Participant #10
	Bento Box lunch box	"I think that's kind of helping them eat their fruits and vegetables more because then if they're not warm, who wants to eat a warm, you know, grapes and carrots and cucumbers and stuff like that, like you don't want that warm." - Participant #8

	“I love that there is a freezer option in it, cause that's something we don't currently have, but yeah, a bit too small for the amount of food that I feel like we need to send our kids sometimes or, you know, or the idea if you want to separate stuff, I need more places to separate, right? But I mean, quality wise, it's very good, very sturdy. I love the ice box part of it.” - Participant #18<br><br>“It's good for a little kid, but as the kids growing, I was thinking I should go for a bigger lunch box which have big portion size. It's good for JK and SK.”-Participant#11
Text messages	“Personally, it just didn't add more value.” - Participant #4<br><br>“I was always paying attention to them, cause, you know, I'm always looking more tips, and I do try and follow whatever I can.” - Participant #10<br><br>“I'm going to be honest; I didn't really look at them.” - Participant #11<br><br>“I think some of them were some that I already knew about, or I was sort of already doing.” -Participant #10<br><br>“I think a lot of them we actually already did follow. So, you know, I think “packing the night before” was one, I can't remember exactly all of them. So, when I read them, I was like “yeah, we're on that already”, so they weren't ground-breaking or anything.” -Participant#18
Family cooking class	“It was actually really nice. I think in person would have been a little different experience and maybe better for like the kids because she didn't quite understand a concept of like watching a video. I know it's not always possible so like I don't, I don't mind but I really liked it just to have like that interaction with other families. I think this is something that could have been done more than once actually.” - Participant #6<br><br>“I felt like I want to be there to just try with you guys and just taste what was cooking in your kitchen maybe… So being in person rather than doing it on the screens, I

		think it would be more fun to be in person." - Participant #9<br><br>"It was super awesome seeing that there was a young individual there cooking with you guys that was really cute and he was so enthusiastic and I really think that helped to engage my son." - Participant #18<br><br>"The cooking class, I was not able to do it because of the time." -Participant#4
	Food records (ease of completion, feedback on potential improvements e.g. incentive, complexity)	"Maybe focusing a bit more on the pictures. That's a bit easier at least than the log. Or maybe find a way like to say to ask the kids to take the pictures for them? So, to give them the responsibility." - Participant #4<br><br>"The word documents are a little wonky sometimes when you have to fill them. So, it's more like a technical aspect. But they were very well explained, and the example was very useful, just to know exactly what you're looking for, for each, like ingredients or food item or whatever. So no, it was very easy actually to use." - Participant #6<br><br>"Was a bit tricky for me because I had to write down the quantities of it, the product name exactly. You know, like sometimes you just give them a cheese pizza, like I gave them cheese pizza and then it says that specifically mentioned the name of the brand, which was kinda tricky for me because sometimes I just get some stuff that's you know on sale. It was a lot of detail, besides that it was fine." - Participant #9<br><br>"I would like to have large incentive. I think it's kind of motivating, I guess. I think right now it was $25 for doing the survey and food records so having a larger incentive would help with filling out that food record." - Participant #9
Perceived impact	Perceived impact on participants' ability to pack healthier lunches for their children	"I am like more confident in my meal preparation." - Participant #1<br><br>"From where I was to where I am? Like yeah, I have to say it inspired me to make different meals and better meals." - Participant #8

Perceived changes in food choices	“I was inspired by the ideas to you know I include like chicken wraps or fish wraps now, before my kids wouldn't even consider fish and my son loves it now.” - Participant #10<br><br>“It definitely made me, like, more aware of what I'm going to put in her lunches, I mean, as much as I can because like I said, I do want her to eat, but it’s so hard to eat. But even like some meal prepping, it kind of, like made me think of that and it did also make me reflect on, like, how important it is to have her help with packing her lunches and making choices. So, you know, we try and now, we're more intentional with grocery shopping and being like ‘OK, [child name], why don't you think of some things that, like, you want in your lunch?’ So, yeah, it made me think about it more.” - Participant #11
